# Genetic and Epigenetic Alterations Induced by Pesticide Exposure: Integrated Analysis of Gene Expression, microRNA Expression, and DNA Methylation Datasets

**DOI:** 10.3390/ijerph18168697

**Published:** 2021-08-17

**Authors:** Federica Giambò, Gian Marco Leone, Giuseppe Gattuso, Roberta Rizzo, Alessia Cosentino, Diana Cinà, Michele Teodoro, Chiara Costa, Aristides Tsatsakis, Concettina Fenga, Luca Falzone

**Affiliations:** 1 CEMAD Digestive Disease Center, Fondazione Policlinico Universitario “A. Gemelli” IRCCS, Università Cattolica del Sacro Cuore, 00168 Roma, Italy; federica.giambo@guest.policlinicogemelli.it; 2 Department of Biomedical and Biotechnological Sciences, University of Catania, 95123 Catania, Italy; g.marco-94@outlook.it (G.M.L.); peppeg9305@gmail.com (G.G.); rroberta342@gmail.com (R.R.); alessia.cosentino@gmail.com (A.C.); 3 Health Management of the “Cannizzaro” Emergency Hospital of Catania, 95126 Catania, Italy; dianacinact@gmail.com; 4 Clinical Pathology and Clinical Molecular Biology Unit, “Garibaldi Centro” Hospital, ARNAS Garibaldi, 95123 Catania, Italy; 5 Department of Biomedical and Dental Sciences and Morphofunctional Imaging, Occupational Medicine Section, University of Messina, 98125 Messina, Italy; michele.teodoro@unime.it (M.T.); concettina.fenga@unime.it (C.F.); 6 Clinical and Experimental Medicine Department, University of Messina, 98125 Messina, Italy; ccosta@unime.it; 7 Department of Forensic Sciences and Toxicology, Faculty of Medicine, University of Crete, 71003 Heraklion, Greece; tsatsaka@uoc.gr; 8 Epidemiology and Biostatistics Unit, National Cancer Institute-IRCCS ‘Fondazione G. Pascale’, 80131 Naples, Italy

**Keywords:** pesticides, microRNAs, DNA methylation, biomarkers, epigenetics, bioinformatics, tumors, neurodegenerative diseases

## Abstract

Environmental or occupational exposure to pesticides is considered one of the main risk factors for the development of various diseases. Behind the development of pesticide-associated pathologies, there are both genetic and epigenetic alterations, where these latter are mainly represented by the alteration in the expression levels of microRNAs and by the change in the methylation status of the DNA. At present, no studies have comprehensively evaluated the genetic and epigenetic alterations induced by pesticides; therefore, the aim of the present study was to identify modifications in gene miRNA expression and DNA methylation useful for the prediction of pesticide exposure. For this purpose, an integrated analysis of gene expression, microRNA expression, and DNA methylation datasets obtained from the GEO DataSets database was performed to identify putative genes, microRNAs, and DNA methylation hotspots associated with pesticide exposure and responsible for the development of different diseases. In addition, DIANA-miRPath, STRING, and GO Panther prediction tools were used to establish the functional role of the putative biomarkers identified. The results obtained demonstrated that pesticides can modulate the expression levels of different genes and induce different epigenetic alterations in the expression levels of miRNAs and in the modulation of DNA methylation status.

## 1. Introduction

Pesticides are a heterogeneous group of chemical compounds widely used in agriculture and livestock to kill pests and weeds [1]. Due to their chemical characteristics, bioaccumulation, and low selectivity, pesticides are classified as toxic substances for other organisms, including humans [2].

Observational studies on workers exposed to pesticides have shown how these chemicals represent an important risk to public health, with about 3 million cases of pesticide poisoning every year [3]. This risk is particularly relevant for individuals who live in agricultural areas, in which pesticides are mainly used, or those occupationally exposed to pesticides [2,4].

A direct link has been well established between the environmental or occupational exposure to pesticides and the development of several chronic-degenerative diseases, including tumors, neurological disorders, and autoimmune diseases [5,6,7]. The pathogenetic mechanisms of pesticides rely on both genetic and epigenetic modifications, affecting key genes and enzymes involved in the molecular mechanisms that underlie the development of neoplastic or neurodegenerative diseases [8].

Although pesticide-associated genetic alterations are well known and characterized [9,10], the epigenetic alterations induced by pesticide exposure and responsible for the development of these diseases have not been fully investigated yet [11].

It should be noted that epigenetics is the study of heritable changes in gene expression that occur without a change in the DNA sequence [12]. Epigenetic alterations are now recognized as early events of disease promotion, including neoplastic transformation; therefore, the analysis of such modifications would allow the early detection of predisposing conditions or preneoplastic lesions in people at high risk for environmental or occupational exposure to pesticides [13]. Among the epigenetics alterations, DNA methylation, histone modifications, and microRNA (miRNA) alterations are the most studied [14,15,16]. In particular, DNA methylation is a covalent modification involving the transfer of a methyl group onto the C5 position of the cytosine to form 5-methylcytosine. It regulates gene expression by recruiting proteins involved in gene repression or by inhibiting the binding of transcription factor(s) to DNA [16].

DNA methylation in a promoter region causes gene silencing, resulting in the down-regulation of the gene itself; on the contrary, intragenic hypermethylation is associated with gene up-regulation [17,18].

Regarding histone modifications, all histones are subject to post-transcriptional modifications, including acetylation, methylation, phosphorylation, ubiquitination, SUMOylation, and ADP-ribosylation [19].

The epigenetic alterations of histones have important roles in transcriptional regulation as histones can regulate the conformation of the DNA structure into actively transcribed euchromatin and transcriptionally inactive highly condensed heterochromatin. The histones forming the euchromatin are characterized by high levels of acetylation, while heterochromatin is generally characterized by low levels of acetylation [20].

Regarding miRNAs, these are small noncoding RNA fragments of 20-22 bp able to bind specific messenger RNAs (mRNAs) by inducing their silencing. In particular, when the complementarity between the miRNA sequence and mRNA target is complete, the RNA-induced silencing complex (RISC) induces the degradation and permanent silencing of the targeted mRNA; otherwise, the RISC complex induces a transient inhibition of gene translation in the case of an imperfect match between miRNA and mRNA. [21].

Some studies have already associated pesticide exposure with the aforementioned epigenetic alterations [8,22,23]; however, to the best of our knowledge, there are no studies that encompass the study of both genetic and epigenetic alterations associated with pesticide exposure. The analysis of both genetic and epigenetic alterations in individuals chronically exposed to such poisoning chemicals could be predictive for the identification of pathological conditions, including tumors and neurodegenerative diseases.

On these bases, the aim of the present study was to identify a panel of genetic and epigenetic alterations induced by the exposure to pesticides that could be predictive for the development of diseases associated with these toxicants. For this purpose, the bioinformatics data reported in the literature and related to both genetic and epigenetic alterations mediated by pesticides were analyzed in order to establish the molecular alterations associated with the exposure to pesticides and predisposing the development of oncological and neurodegenerative diseases.

## 2. Materials and Methods

### 2.1. Selection of Gene Expression, miRNA Expression, and DNA Methylation Datasets

The Gene Expression Omnibus (GEO) DataSets database, publicly available on NCBI (www.ncbi.nlm.nih.gov/geo/, accessed on 15 August 2021), was used to select the DNA methylation, gene expression, and miRNA expression datasets of individuals with and without pesticide exposure.

In order to evaluate the impact of pesticides only in humans, an advanced search using the following search terms was performed: ‘((pesticides) AND ‘Homo sapiens’ [porgn: txid9606]).’ In this way, only datasets containing the expression or methylation data from individuals or human cells exposed to pesticides were collected.

The selected datasets included datasets published up to February 2020 using the following inclusion and exclusion criteria to select only relevant datasets:

Inclusion criteria: (i) datasets containing expression data on human samples, human cell lines, or animal models investigating potential biomarkers for humans; (ii) case-control studies, with cells or individuals exposed or not to pesticides.

Exclusion criteria: (i) toxicity studies of pesticides in animals or vegetables, studies conducted on human cell lines or in vivo with less than 10 samples; (ii) datasets with genes, methylation probesets, or miRNAs not correctly annotated.

### 2.2. Differential Analyses and Selection of Candidate Genetic and Epigenetic Biomarkers

After selecting and downloading the methylation, gene expression, and miRNA expression data matrices from GEO DataSets, differential analyses were performed between exposed and nonexposed samples to identify genetic and epigenetic factors dysregulated by pesticides. For this purpose, the GEO2R bioinformatic tool available on the GEO DataSets database was used. In particular, GEO2R allows the clustering of the samples of each dataset into a ‘nonexposed control group’ and ’exposed samples group,’ performing the differential analysis between expression levels of each gene, miRNA, and methylation hotspot in every dataset. Thus, it was possible to identify a panel of up-regulated or down-regulated genes, miRNAs, as well as hypo- and hyper-methylated hotspots altered as a result of exposure to different pesticides.

### 2.3. Functional Role of the Candidate Genetic and Epigenetic Biomarkers

In order to better understand the modulating effects of pesticides on the expression levels of miRNAs, as well as to predict the functional role of the candidate genetic and epigenetic biomarkers previously identified, several bioinformatic analyses were performed to identify the gene molecular pathways actively modulated by the selected miRNAs. For this purpose, first, bioinformatic analysis was performed by using the miRTargetLink tool, as previously described [24]. This software allows one to obtain detailed information about miRNA–mRNA interactions, analyzing the data stored on different public databases of miRNA expression and their interaction with target genes [25]. In addition, DIANA-miRPath v.3, a computational pathway prediction tool, was used to identify the cellular and molecular pathways altered by the previously identified miRNAs [26].

Subsequently, two bioinformatics software, STRING v11.0 (https://string-db.org/, accessed on 13 July 2021) and GO Panther v14.0 (http://pantherdb.org/, accessed on 13 July 2021), were used to analyze the interaction network and the biological and cellular roles of the genes identified through GEO2R, the genes targeted by the selected miRNAs, and the genes with alterations in the DNA methylation levels. In particular, STRING analysis was used to establish the molecular link between differentially expressed genes. The same analysis was performed on hypo- and hyper-methylated genes after pesticide exposure and genes targeted by the differentially expressed miRNAs [27]. Then, GO Panther analysis was performed to establish the functional role of the identified molecular markers related to their molecular function, biological process, protein class, and molecular pathway [28]. These analyses allowed us to better understand the association between the identified genetic and epigenetic markers and the development of oncological or neurodegenerative diseases associated with pesticides exposure.

### 2.4. Statistical Analyses

The fold change values obtained through the differential analyses performed by using GEO2R software were expressed as log2FC. Differentially expressed genes, miRNAs, and methylation hotspots were considered significantly altered with a *p*-value of *p* < 0.05. All the data obtained from GEO DataSets were already normalized by the GEO2R software [29].

With regard to the functional prediction analyses, DIANA-mirPath, STRING, and GO Panther automatically apply proper normalization and statistical evaluations, providing significant data expressed as *p* < 0.05.

## 3. Results

### 3.1. Selection of Gene Expression, miRNA Expression, and DNA Methylation Datasets and Identification of Potential Genetic and Epigenetic Biomarkers of Pesticide Exposure

By using the aforementioned search terms, 32 different datasets were identified on the GEO DataSets database. Most of these datasets included gene expression data obtained from different biological matrices, human cell lines, and animal models after pesticide exposure, while only a small number contained data about miRNA expression or DNA methylation. Despite the identification of these 32 datasets, most of them did not satisfy the pre-established inclusion and exclusion criteria, due to the small number of samples or due to the incorrect annotation of genes or methylation probesets. 

Therefore, using the inclusion and exclusion criteria mentioned above, it was possible to select nine different datasets. Six contained gene expression data, two datasets contained methylation data, and only one dataset contained data about miRNA expression after pesticide exposure. The main characteristics of the selected datasets are shown in Table 1.

Due to the limited number of datasets available, data obtained from cell lines treated with pesticides, animal models treated with similar substances, and individuals with professional or environmental exposure to pesticides were evaluated. In addition, different classes of pesticides have been taken into account to evaluate the global genetic and epigenetic effects induced by these chemicals. Among the pesticides taken into account, there were atrazine, imazalil, fenbuconazole, 2,4-D, arsenic trioxide, triadimefon, cyproconazole, rotenone, and others (Table 1).

**Figure 1:**
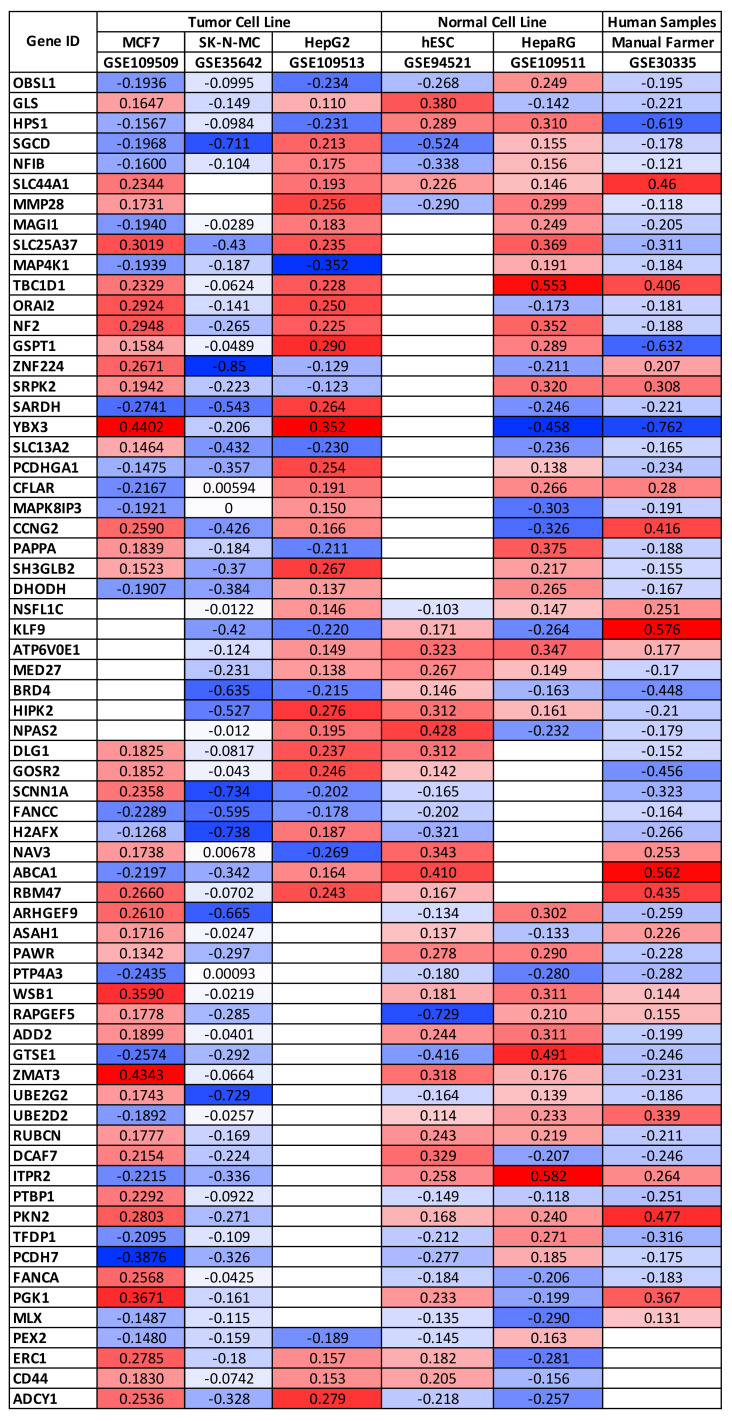
Differentially expressed genes after pesticide exposure in human samples and in vitro models. The fold change values are expressed as log2 of fold change. Up-regulated genes are highlighted with red scale boxes while down-regulated genes are highlighted with blue scale boxes.

The differential analyses performed on the gene expression datasets allowed the identification of a list of differentially expressed genes for each of the six selected datasets. In particular, a list of differentially expressed genes ranging from 1730 to 4785 was obtained. By merging these six lists of genes, it was possible to identify a set of 66 genes significantly up-regulated or down-regulated as a consequence of pesticide exposure in at least five out of six datasets selected (Figure 1).

Interestingly, only two genes, SLC44A1 and FANCC, showed concordant expression levels in all of the six datasets analyzed being up-regulated and down-regulated, respectively. Contrariwise, the other genes showed a certain heterogeneity in their expression levels observed within each dataset. In detail, all the other genes were up-regulated or down-regulated depending on the type of cell line considered, if normal or tumoral. Indeed, it is possible to observe a greater gene expression concordance within the three tumor cells (MCF7, SK-N-MC, and HepG2), as well as within the two normal cell lines (HepRG and hESC), together with samples obtained from individuals professionally exposed to pesticides (GSE30335).

It should be noted that the GSE35642 datasets containing gene expression data obtained from neuroblastoma cell lines exposed or not to pesticide reported a significant down-regulation for all of the 66 genes identified. A possible explanation of these data may rely on the type of cells but may also be due to the type of pesticide tested (rotenone) and the microarray platform used by the authors.

These first analyses have allowed the identification of a panel of genes significantly modulated by different pesticides. In addition, these results revealed that pesticides are able to alter the expression levels of the same genes in different histological contexts; however, the effects of pesticides are different according to the type of cells (normal or tumor cells). A further selection of dysregulated genes was also performed, selecting only the dysregulated genes with concordant expression levels in the three datasets of normal cells and the two datasets of tumor cells; however, this further selection criterion implicated a significant loss of data; therefore, the subsequent analyses were carried out on the 66 genes shown in Figure 1 and Figure S1.

**Figure 2:**
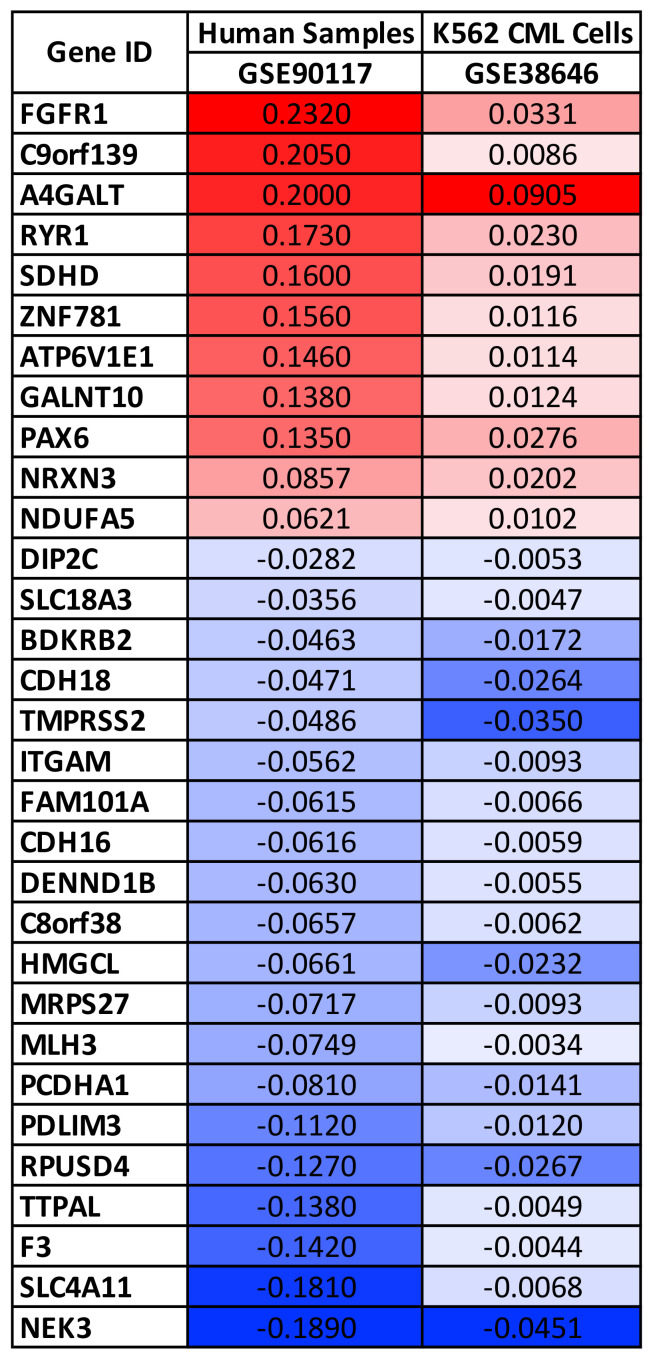
Differential gene methylation status after pesticide exposure. The fold change values are expressed as log2 of fold change. Hyper-methylated genes are highlighted with red scale boxes while hypo-methylated genes are highlighted with blue scale boxes.

In the same manner, the differential analyses performed for the two datasets containing DNA methylation data have allowed the identification of a panel of 62 genes with differential methylation patterns as a consequence of pesticides exposure. Of these 62 genes, 31 showed concordant methylation alterations as they were hypermethylated or hypomethylated in both datasets (Figure 2).

The differential analyses revealed that the highest methylated genes were FGFR1 and A4GALT, while the lowest methylated ones were TMPRSS2, SLC4A11, and NEK3 (Figure 2).

Subsequently, differential analyses were performed for the exposed and nonexposed samples to identify miRNAs dysregulated by pesticides. Unlike what was described for the analysis of the gene expression and methylation datasets, the detection of dysregulated miRNAs was performed only in one dataset, GSE78805. The analysis revealed a total of 20 significantly dysregulated miRNAs (*p* < 0.05) after atrazine exposure, of which five miRNAs were up-regulated and 15 miRNAs down-regulated. In particular, the miRNAs hsa-miR-3149 and hsa-miR-4505 were the most up-regulated, while the miRNAs hsa-miR-10b-3p, hsa-miR-4515, hsa-miR-4313, and hsa-miR-126-3p showed the highest rate of down-regulation (Table 2).

Overall, the differential analyses performed for the gene expression, miRNA expression, and DNA methylation datasets have allowed the identification of both genetic and epigenetic hallmarks of pesticide exposure that could also be used to predict disease susceptibility.

### 3.2. Identification of Genes and Pathways Modulated by the Selected miRNAs

**Figure 3:**
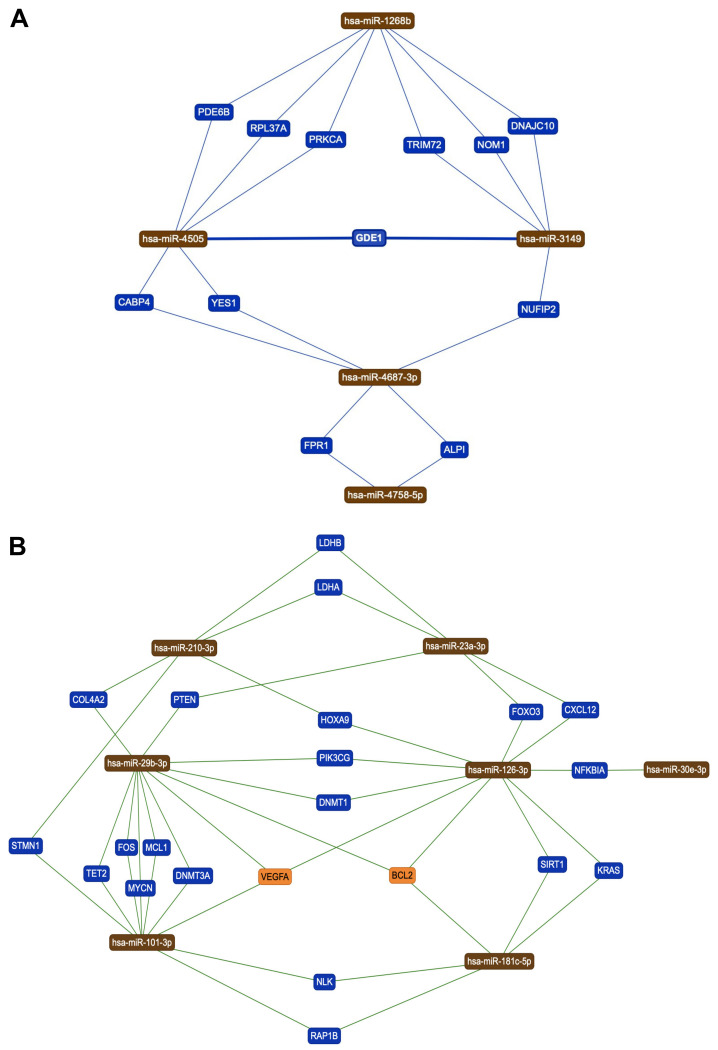
(**A**) miRNA–mRNA interaction network for the five miRNAs up-regulated after pesticide exposure; (**B**) miRNA–mRNA interaction network for the 15 miRNAs down-regulated miRNAs after pesticide exposure.

In order to identify the genes directly modulated by the 20 miRNAs altered by pesticide exposure, the miRTargetLink Human prediction tool was used. This analysis allowed the identification of genes targeted at least by two of the 20 miRNAs identified. More in detail, two independent miRTargetLink Human analyses were performed, one for the up-regulated miRNAs and another for the down-regulated ones. With regard to the five up-regulated miRNAs after pesticide exposure, these bind 12 different genes in an integrated fashion, precisely the PDE6B, RPL37A, PRKCA, TRIM72, NOM1, DNAJC10, GDE1, CABP4, YES1, NUFIP2, FPR1, and ALPI genes (Figure 3A).

In the same manner, the miRTargetLink Human analysis performed for the 15 miRNAs down-regulated after pesticide exposure revealed more complex miRNAs–genes integrated networks consisting of seven hub miRNAs (hsa-miR-210-3p, hsa-miR-23a-3p, hsa- miR-29b-3p, hsa-miR-126-3p, hsa-miR-30e-3p, hsa-miR-101-3p, and hsa-miR-181c-5p) targeting 22 different genes (LDHB, LDHA, COL4A2, PTEN, FOXO3, CXCL12, HOXA9, PIK3CG, DNMT1, STMN1, TET2, FOS, MCL1, DNMT3A, MYCN, VEGFA, BCL2, SIRT1, KRAS, NFKBIA, NLK, and RAP1B) (Figure 3B).

It should be noted that some of these genes were targeted by three different miRNAs, particularly VEGFA and BCL2, suggesting how pesticide exposure might strongly modulate the expression of these genes through epigenetic mechanisms mediated by miRNAs. Interestingly, among the 22 genes targeted by the seven down-regulated miRNAs, there were genes notoriously involved in the development and progression of different diseases such as PTEN, KRAS, VEGFA, BCL2, FOS, DNMT1, MCL1, DNMT3A, and FOXO3. In addition, these data also suggested that pesticide exposure can profoundly alter human epigenetics through the alteration of miRNAs and, in turn, through the modulation of genes involved in DNA methylation such as DNMT1 and DNMT3A.

After identifying the two miRNAs–genes interaction networks, a second prediction analysis was performed to identify the molecular pathways directly modulated by the 20 selected miRNAs. For this purpose, the DIANA-miRPath v.3 software was used to predict the pathways modulated by the five up-regulated and the 15 down-regulated miRNAs following pesticides exposure.

For the five up-regulated miRNAs, the DIANA-miRPath analysis revealed eight different modulated pathways and a total of 75 modulated genes, including some genes identified through the previous miRTargetLink Human analysis (e.g., PRKCA) (Table 3).

Table 3 shows that the five up-regulated miRNAs were involved in the regulation of signal transduction pathways, and hormone and neurocognitive pathways, suggesting how pesticide exposure might be involved in the development of both tumor and neurodegenerative diseases through the alteration of these pathways.

Similarly, the DIANA-miRPath analysis allowed the identification of genes and related pathways modulated by the 15 miRNAs down-regulated after exposure to pesticides. In this latter case, only nine of the 15 down-regulated miRNAs showed interaction with specific pathways. Overall, these miRNAs were able to target a total of 3096 genes and 62 different molecular pathways. Of these 62 pathways, ten were related to infectious diseases and, therefore, were not directly correlated with pesticide exposure. Thus, Table 4 shows the 52 pathways targeted and modulated by the selected miRNAs that are involved in both tumor development (e.g., proteoglycans in cancer (hsa05205), PI3K-Akt signaling pathway (hsa04151), Pathways in cancer (hsa05200), and Transcriptional misregulation in cancer (hsa05202)), hormonal imbalance (e.g., estrogen signaling pathway (hsa04915), neurotrophin signaling pathway (hsa04722), and thyroid hormone signaling pathway (hsa04919)), and neurodegenerative diseases (e.g., neurotrophin signaling pathway (hsa04722), and Axon guidance (hsa04360)) (Table 4).

DIANA-miRPath analysis demonstrated that almost all of the selected miRNAs were strongly involved in the modulation of several cellular and molecular pathways underlying the development of several pathologies.

**Figure 4:**
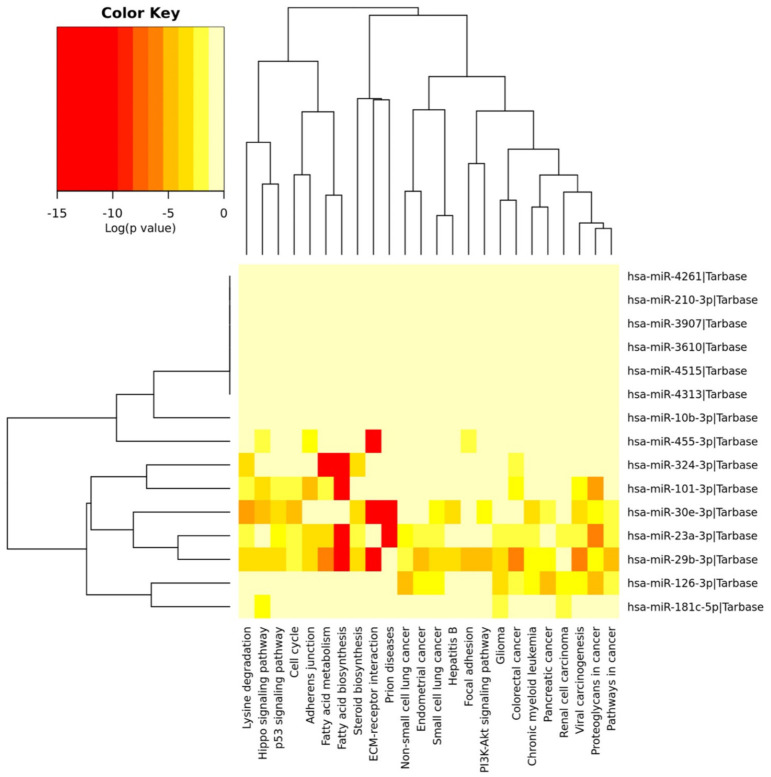
Dendrogram showing the interactions between deregulated miRNAs and major altered pathways.

Due to the high number of genes and pathways modulated by the down-regulated miRNAs, it was also possible to obtain a dendrogram summarizing the strongest interaction between the 15 down-regulated miRNAs and the pathways identified (Figure 4).

### 3.3. Protein-Protein Interaction and Functional Role of the Epigenetic Biomarkers and the Differentially Expressed Genes Identified after Pesticide Exposure

To further unveil the effects of both genetic and epigenetic alterations induced by pesticide exposure, the protein-protein interaction (PPI) and functional role of the identified putative biomarkers were assessed by using STRING and GO Panther bioinformatics tools, respectively.

**Figure 5:**
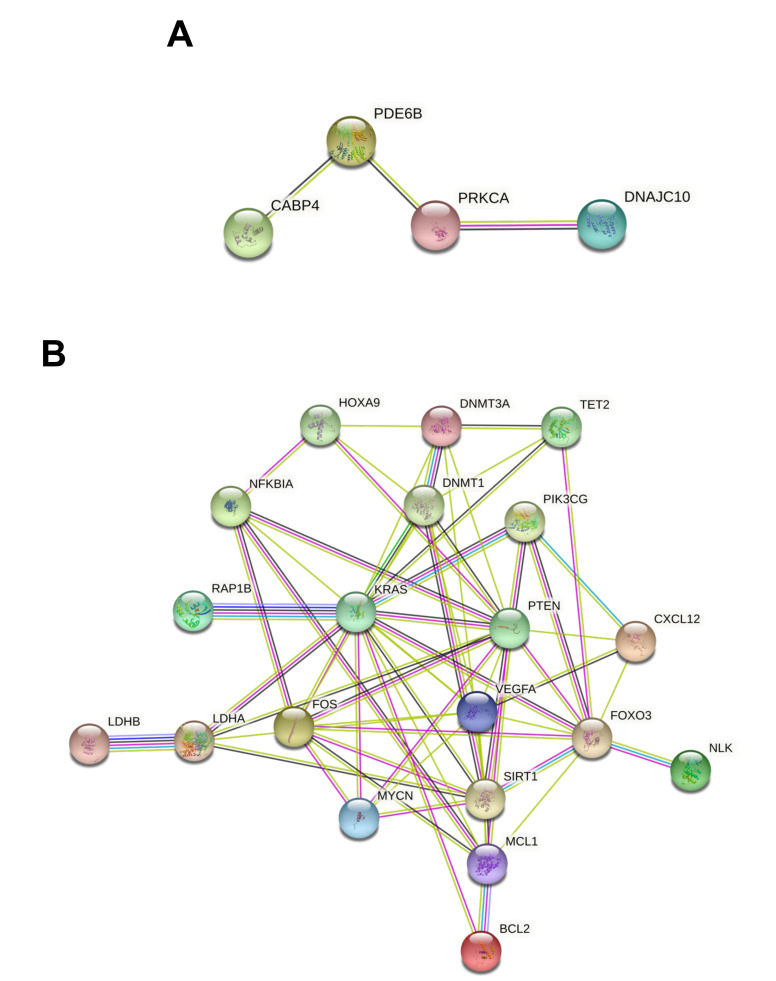
(**A**) STRING PPI network observed for the 12 genes altered by the five up-regulated miRNAs after pesticide exposure; (**B**) STRING PPI network observed for the 22 genes altered by the seven up-regulated miRNAs after pesticide exposure.

First, the PPI network of the genes targeted by the five up-regulated miRNAs identified through miRTargetLink Human was identified. Of the 12 genes previously identified, only four were interconnected. Despite the low number of gene interactions, this further analysis highlighted again how the PRKCA gene might be involved in pesticide-associated molecular alterations, as suggested by the miRTargetLink Human and DIANA-miRPath analyses (Figure 5A). In particular, this gene was directly related to DNAJC10 and PDE6B.

A more complex PPI network was obtained by analyzing the 22 genes modulated by seven of the 15 down-regulated miRNAs, as predicted by miRTargetLink Human analysis. In this case, STRING analysis revealed that 20 out of 22 genes were involved in an integrated PPI network (Figure 5B). In particular, both the number and interaction levels were stronger compared to those observed in the previous string analysis (Figure 5A). Among these 20 interconnected genes, hub genes were KRAS, PTEN, FOXO3, and VEGFA, which established numerous connections with multiple genes. Of note, these genes are recognized as key factors involved in tumor development when dysregulated. STRING analysis also revealed that DNMT3A and DNMT1, involved, respectively, in the de novo DNA methylation and in the maintenance of the methylation status, were strongly interconnected with the aforementioned genes, thus contributing to their alteration. Similarly, SIRT1, responsible for the deacetylation of the proteins involved in the regulation of the cellular response to stress and longevity, showed different interactions both with proteins involved in DNA methylation and with genes involved in the alteration of intracellular signaling (Figure 5B).

This first STRING analysis, performed on the genes altered by the up-regulated and down-regulated miRNAs associated with pesticide exposure, highlighted how pesticides could induce a strong epigenetic modulation in key genes involved in different pathological processes. Therefore, the evaluation of both miRNAs and targeted gene expression levels could be used for diagnostic purposes to predict the degree of exposure to these toxic substances and consequently predict the risk of the onset of pathologies related to prolonged exposure to pesticides.

To further uncover the functional role of the genes altered by the selected miRNAs, GO Panther enrichment was performed. The GO Panther analysis conducted on genes altered by up-regulated and down-regulated miRNAs showed that they are all firmly involved in different molecular functions, biological processes, and molecular pathways. For all the genes identified, the type of coding protein was highlighted to better understand its action at the cellular and molecular levels.

**Figure 6:**
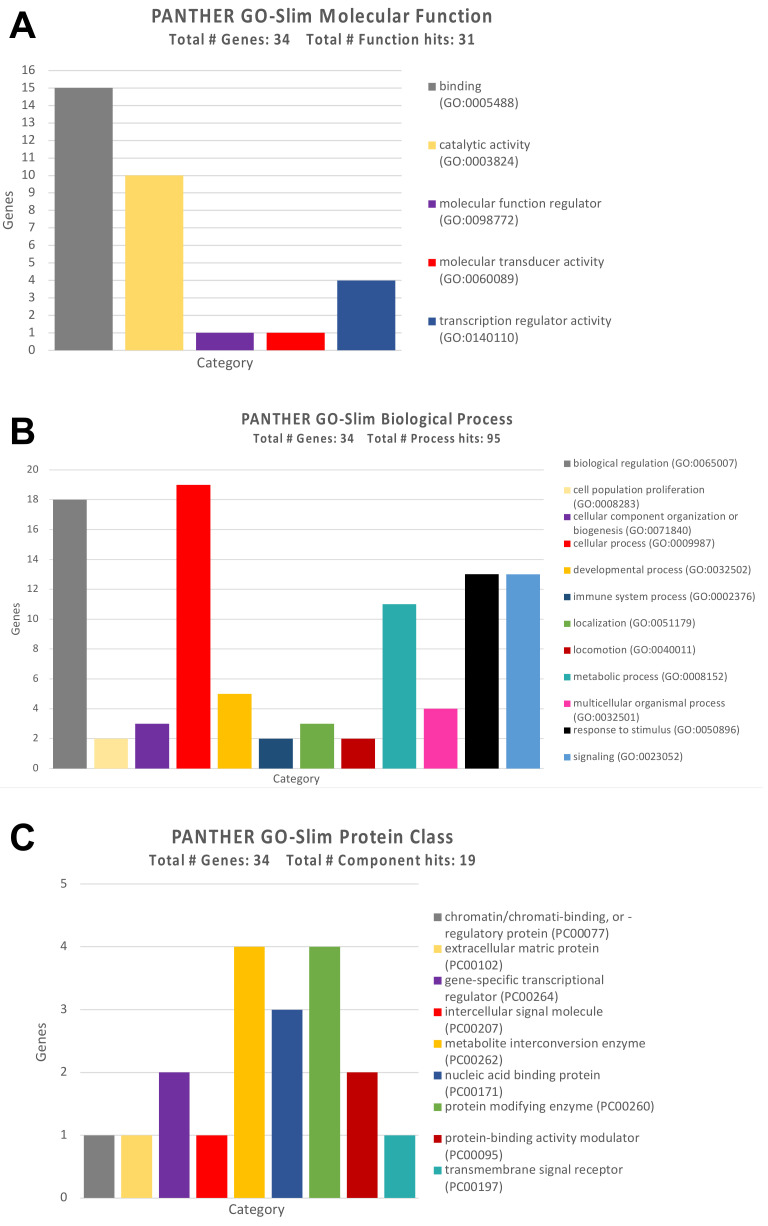
(**A**) GO Panther analysis of the molecular functions performed by the genes modulated by the identified miRNAs; (**B**) GO Panther analysis of biological processes in which the genes modulated by the identified miRNAs are involved; (**C**) GO Panther analysis of the protein classes of the genes modulated by the identified miRNAs.

As for the molecular functions, the GO Panther analysis has highlighted how almost 50% of the identified genes (15 genes) were involved in the molecular binding of other proteins, while a large part of them, ten genes, were involved in fundamental cellular catalytic activities (Figure 6A).

The same analysis performed for the ‘Biological Process’ category revealed that the majority of genes were involved in cellular processes and in the regulation of various biological processes, including cell communication, cell death, and cell homeostasis (Figure 6B).

Regarding the type of proteins codified by the previously identified genes, these were mainly enzymes involved in the cell metabolisms and detoxification, as well as protein-modifying enzymes such as SIRT1 and DNA methyltransferases (Figure 6C).

The same STRING and GO Panther analyses were also performed for the methylation and genetic markers previously identified by differential analysis.

**Figure 7:**
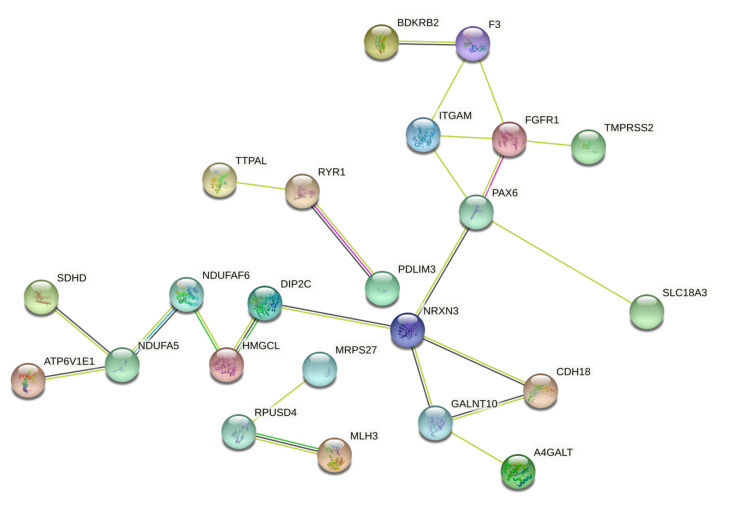
STRING PPI network observed for the 31 genes with altered methylation status after pesticide exposure.

Regarding the PPI network between the 31 methylated and demethylated genes, the STRING analysis showed how these genes form a moderate network where NRXN3 and PAX6 genes, both hypermethylated after pesticide exposure, represented two key nodes (Figure 7). These two genes are fundamental for neuronal and neurovisual development; therefore, these data suggest how epigenetic modifications associated with chronic pesticide exposure could be responsible for the development of nervous dysfunctions, especially for the unborn if exposure occurs during the first weeks of intrauterine life.

In order to establish the functional role of this second panel of genes, the Gene Ontology analysis allowed us to establish the biological processes, molecular functions, and protein classes of the 31 genes with an altered DNA methylation status.

**Figure 8:**
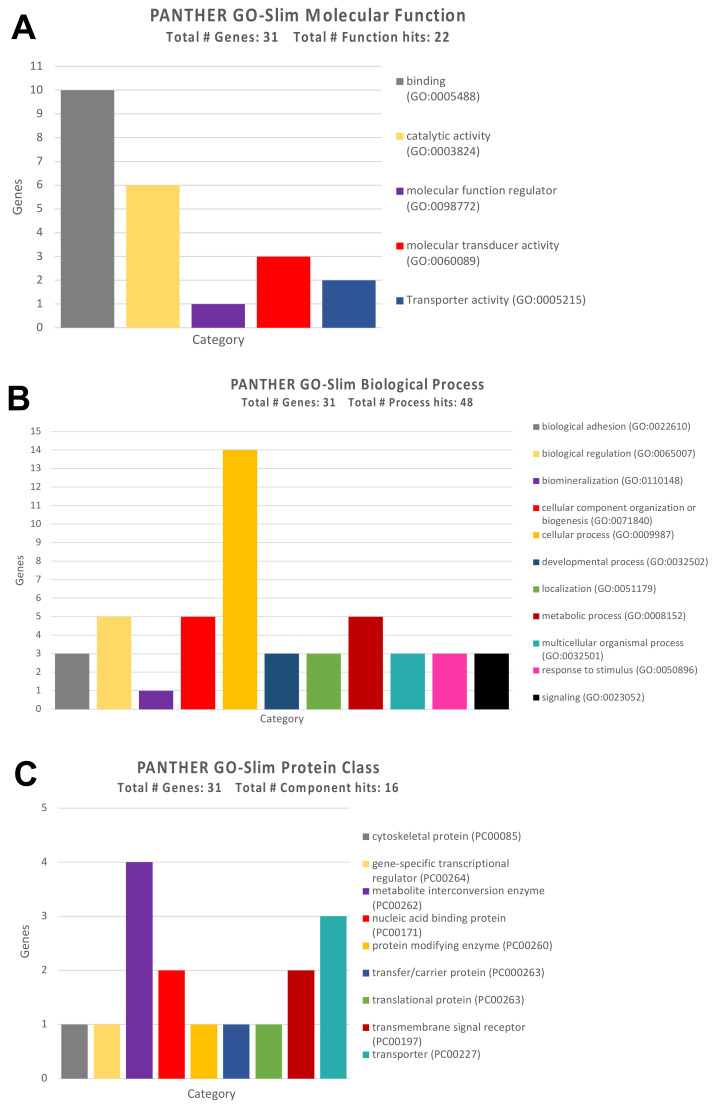
(**A**) GO Panther analysis of the molecular functions performed by the differentially methylated genes after pesticide exposure; (**B**) GO Panther analysis of biological processes in which the differentially methylated genes after pesticide exposure are involved; (**C**) GO Panther analysis of the protein classes of the differentially methylated genes after pesticide exposure.

Similar to what was observed for the genes targeted by the selected miRNAs, pesticide-associated demethylated genes were mainly involved in the molecular functions of protein binding (but also binding with ions, lipids, and organic compounds), and in some catalytic functions mainly represented by redox reactions (Figure 8A).

Analyzing the functional category of biological processes, GO Panther analysis highlighted a strong grouping of demethylated genes involved in different cellular processes and, in particular, cellular metabolic processes, the organization of cellular components, and the regulation of various biological functions (Figure 8B). In addition, the demethylated genes belong mainly to the protein class of the metabolite interconversion enzymes, transport proteins, and DNA binding proteins (Figure 8C).

**Figure 9:**
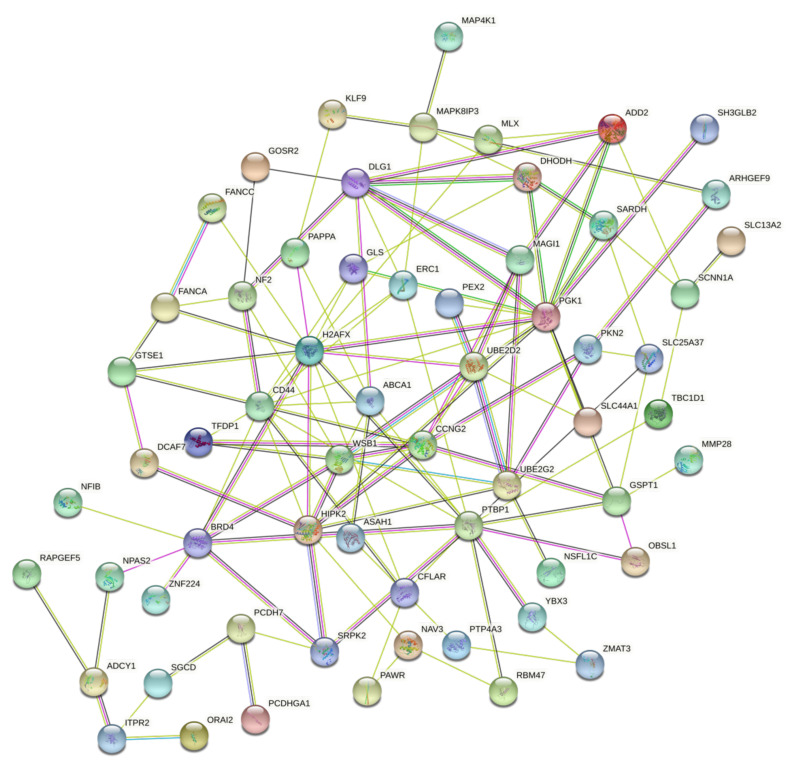
STRING PPI network observed for the 66 differentially expressed genes after pesticide exposure in the 6 datasets analyzed.

Finally, the last step of the analysis concerned the evaluation of the PPI network and of the functions of genes differentially expressed due to exposure to pesticides. Therefore, in this last phase of the analysis, the 66 genes differentially expressed in the six gene expression datasets analyzed were considered. The STRING analysis showed a rich network of interactions between the dysregulated genes. These data appear much more significant if we consider that these genes were identified by analyzing six different datasets and considering only the genes with a *p*-value < 0.01 (Figure 9).

In this latter case, it is possible to identify hub genes strongly interconnected with the identified genetic biomarkers; among these hub genes, PGK1, DLG1, PTBP1, HIPK2, H2AFX, etc. were able to bind different genes involved in different cellular processes such as genes belonging to the MAPK pathway (MAP4K1 and MAPK8IP3), receptor molecules and proteins involved in the degradation of the extracellular matrix (CD44, MMP28, etc.), and proteins with several ontologies.

**Figure 10:**
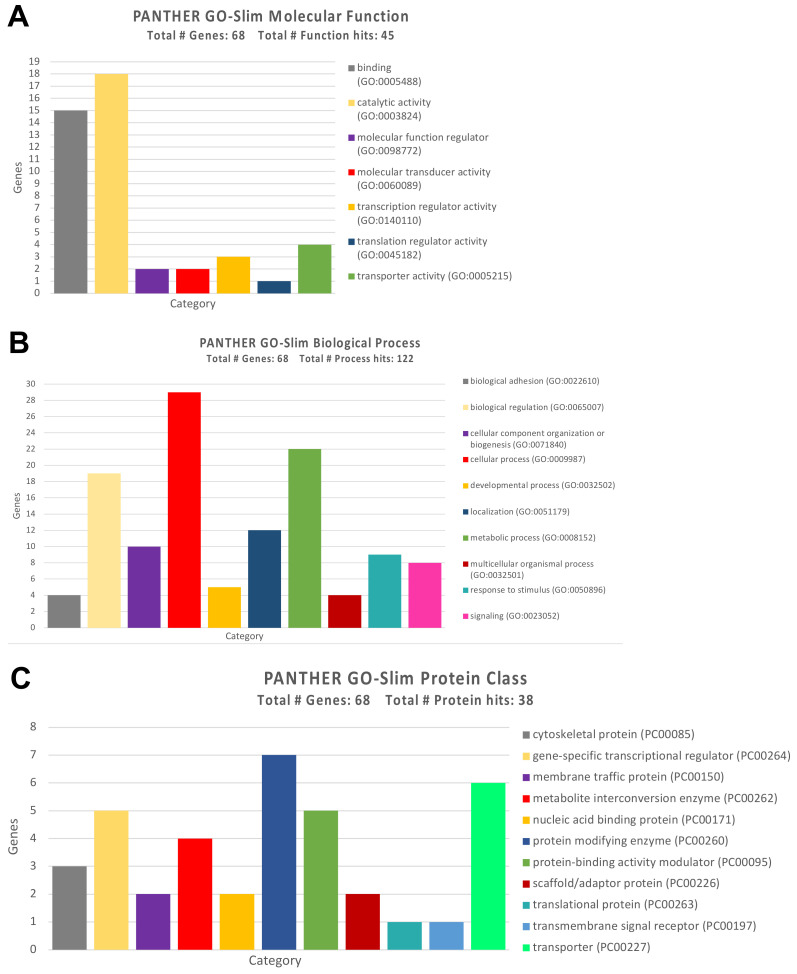
(**A**) GO Panther analysis of the molecular functions performed by genes differentially expressed after pesticide exposure; (**B**) GO Panther analysis of the biological processes in which the differentially expressed genes are involved; (**C**) GO Panther analysis of the protein classes of the differentially expressed genes after pesticide exposure.

With regard to the molecular functions, the 66 differentially expressed genes were mainly involved in catalytic activities (hydrolases and transferases) and in the binding of proteins, organic heterocyclic compounds, and organic cyclic compounds (Figure 10A).

The subsequent analysis of the ‘Biological Process’ category highlighted a strong prevalence of genes involved in the metabolic and biosynthesis cellular process, suggesting how the alteration of these genes induced by pesticides could be related to the incomplete detoxification of xenobiotics with the consequent harmful accumulation of toxic metabolites for the cells (Figure 10B).

The analysis of ‘Protein Class’ has demonstrated that most of the 66 dysregulated genes were protein-modifying enzymes, translational proteins, and transcriptional regulators (Figure 10C).

These further bioinformatic analyses allowed us to establish the functional roles of the genetic and epigenetic biomarkers predictive for pesticide exposure and potentially used as early diagnostic biomarkers for both tumor and neurodegenerative diseases.

## 4. Discussion

The integrated bioinformatics analyses of gene expression, miRNA expression, and DNA methylation datasets performed here have allowed the identification of genetic and epigenetic alterations associated with pesticide exposure that could be predictive for the development of both tumor and neurodegenerative diseases. Several studies have demonstrated that environmental and occupational exposure to pesticides represents a significant risk factor for the development of neurodegenerative, neoplastic, immunological, and hormonal disorders [35,36,37,38,39]. In the present study, the attention was focused on both genetic and epigenetic alterations, highlighting how the modulation of DNA methylation status and the over-expression or silencing of miRNAs are associated with the alteration of key cellular and molecular pathways responsible for the development or progression of different human diseases.

Besides the epigenetic alterations, gene modulation after pesticide exposure was also observed, highlighting how the effects of these toxic substances differ depending on whether pesticide exposure occurs in tumor cell lines, normal cell lines, or biological samples obtained from individuals professionally exposed to pesticides.

The results obtained demonstrated that 20 miRNAs were significantly altered by pesticide exposure, of which five were up-regulated and 15 were down-regulated. Among these miRNAs, hsa-miR-210-3p is of particular interest as it has already been associated with the development of Parkinson’s disease, associated with exposure to pesticides and other toxic substances. In particular, Zhang and colleagues (2017) demonstrated that the alteration of the expression levels of hsa-miR-210-3p contributes to the dopaminergic damage observed in meperidine (MPTP)-induced Parkinson’s disease [40]. Other studies have shown that hsa-miR-210 is also involved in the detoxification of xenobiotic substances and in the regulation of the cellular redox state [41]. Similarly, hsa-miR-126 and hsa-miR-29b have been previously associated with pesticides. Specifically, Yuan and colleagues (2018) demonstrated that the serum levels of these two miRNAs were significantly altered in a large series of individuals subject to acute organophosphorus poisoning compared to a group of individuals not exposed to these substances [42]. Among the miRNAs identified here, hsa-miR-181c-5p has also already been associated with the development of Parkinson’s disease. Indeed, it was recently demonstrated that this miRNA is significantly dysregulated in both blood samples and in substantia nigra samples obtained from patients with Parkinson’s disease [43]. In the study of Wirbisky and colleagues (2016), the authors highlighted how the miRNAs identified here are involved in the pathogenesis of neoplastic, neurodegenerative, and vascular diseases [34]. All these studies showed a direct association between pesticide exposure, miRNA alteration, and the development of neurodegenerative diseases.

Other studies demonstrated an indirect involvement of the identified miRNAs in different tumors and neurological diseases, suggesting how the alteration of specific miRNAs induced by pesticides may be associated with an increased risk of disease onset. In this context, hsa-miR-210 and hsa-miR-29 have been associated with the development of breast cancer and uveal melanoma [24,44,45]. In addition, hsa-miR-29 is also involved in developing glioblastoma and Alzheimer’s disease when down-regulated, suggesting its strong involvement in neurological disorders [46,47]. With regard to the involvement of the identified miRNAs in tumor development, other studies have highlighted how miR-101 and miR-30 are associated with the development of mesothelioma and bladder cancer as a consequence of natural fibers and environmental pollution, respectively [36,48]. Finally, several miRNAs identified here are also associated with the development of onco-hematological malignancies and lymphomas [49].

Relevant results were also obtained by analyzing the miRNA–mRNA interaction. It was observed how the selected miRNAs were able to modulate numerous genes involved in the development of tumors, in the detoxification of harmful substances, and in the neurodevelopment and neurotransmission. Among the miRNA-targeted genes, PRKCA appears one of the most modulated. This gene is sensitive to the action of various pesticides, insecticides, and herbicides that, when altering its expression, can cause neurotoxic damages [50].

Other fundamental genes targeted by the selected miRNAs and strongly involved in the epigenetic alterations predisposing the development of tumors are DNMT1 and DNMT3, responsible for the alteration of the DNA methylation status, and VEGFA, BCL2, KRAS, SIRT1, and FOXO3, involved in different physio-pathological processes. In particular, it was demonstrated that VEGFA expression is increased by hexachlorobenzene favoring angiogenesis in breast cancer models [51]. Similarly, pesticide exposure can alter the expression levels of SIRT1 and FOXO3 known to be functionally linked. These two genes are strongly modulated by pesticide exposure; indeed, FOXO3 is involved in the reduction of oxidative stress after paraquat or other pesticide exposure [52], while pesticide exposure can induce the over-expression of SIRT1, which, in turn, increases the demethylation activity toward different proteins [53]. With regard to BCL2, it has been widely demonstrated that the exposure to pesticides can cause breaks and translocations of this gene, mainly represented by the t(14;18) translocation, predisposing the development of lymphomas [37]. In the same manner, pesticide exposure can induce different genetic damages, affecting the KRAS gene associated with an increased risk of bladder cancer [54].

All these data about the impact of pesticide exposure on these genes are strongly supported by the results of the STRING and DIANA-miRPath prediction analyses performed here.

It should be noted that a single dataset on miRNA dysregulation after pesticide exposure was available. In addition, the data generated in the dataset GSE78805 were related to zebrafish larvae treated with atrazine as a single toxicant. However, miRNAs are highly conserved between species, and different studies have demonstrated the validity of zebrafish as an optimal model to study the alterations of human miRNA expression after exposure to toxic agents [55,56,57]; the results obtained here must necessarily be validated in more rigid and well-defined experimental contexts as other miRNAs could be dysregulated by the exposure to other pesticides.

With regard to the data obtained from the analysis of the DNA methylation datasets, these allowed the identification of genes with an altered methylation status suggestive of pesticide exposure. Among the most hyper- and hypo-methylated genes, there were RYR1 and PAX6, already associated with pesticide exposure. In particular, RYR1 is a protein constituting the muscle calcium release channel, and the pesticide-induced methylation affecting its intron is associated with a lower gene expression and consequently with alterations of neurotransmission and muscle contraction [58]. Regarding PAX6, polychlorinated biphenyls can determine an alteration in the methylation status of the whole gene whose dysregulation is associated with chronic B lymphocytic leukemia and the alteration of nerve tissue differentiation [59].

The evaluation of the modulation of gene expression after pesticide exposure has generated heterogeneous results, whose interpretability could be complex. Overall, the analysis of the gene expression datasets revealed that pesticides have different modulating effects on the same genes depending on the cell type examined. In particular, the expression levels of genes were concordant among the group of cancer cells and the group of normal cells, independently, while a general inverse pattern of expression was observed comparing the expression levels of the same genes in the groups of cancer and normal cells. For both normal and cancer cells, the PPI and Gene Ontology analyses have shown once again the existence of a close network of interaction between the various genes that are significantly dysregulated following pesticide exposure. These further analyses have highlighted how pesticide exposure might influence different cellular mechanisms, including DNA mismatch repair, extracellular matrix degradation, and the alterations of signal transduction pathways, all mechanisms involved in the development and progression of different diseases, including tumor and neurodegenerative disorders [60,61,62,63]. However, novel and wider studies performed on in vitro and animal models, as well as in exposed individuals, are mandatory in order to establish the genetic and epigenetic impact of specific compounds and their pathogenetic role for humans. Indeed, the results achieved are merely exploratory and give only a global overview of the genetic and epigenetic alterations associated with harmful exposure to pesticides. In addition, some limitations afflict our study. These are mainly represented by the analysis of independent datasets that are very heterogeneous with each other, containing molecular data relating to tumor and normal human cells, animal models, and human biological samples. Another limitation is represented by the lack of data relating to the molecular effects of individual pesticides. Consequently, the results obtained do not allow us to establish with absolute certainty which genetic or epigenetic alteration is associated with exposure to a specific pesticide. Therefore, further well-conceived validation experiments should be performed.

In this context, the analysis of circulating biomarkers, including circulating DNA alterations, miRNAs, and lncRNA, [64,65,66,67] and the use of novel high-sensitivity and high-throughput technologies [68,69,70] will allow the early identification of both genetic and epigenetic aberrations in individuals at risk for pesticide exposure.

These further analyses will make it possible to accurately identify the iatrogenic power of pesticides and to identify novel candidate biomarkers for the prediction of occupational or environmental pesticide exposure and for pesticide-related oncological and neurodegenerative diseases.

## 5. Conclusions

To the best of our knowledge, this is the first study assessing both the genetic and epigenetic alterations induced by pesticides. Through the computational analysis of gene expression, miRNA expression, and DNA methylation datasets obtained from the GEO DataSets database, key genetic and epigenetic alterations associated with pesticide exposure were identified. In addition, the functional role of the genetic and epigenetic factors identified here was further elucidated by using different prediction analysis tools. Overall, the present study provides a comprehensive overview of the molecular effects induced by a group of pesticides. It should be noted that the results obtained here are only exploratory and show in broad terms the pathways and genetic and epigenetic factors significantly modulated by pesticides; therefore, our findings represent the starting point for further studies that will be performed on a large number of in vitro and in vivo models, as well as exposed individuals, to investigate the molecular alteration induced by a specific pesticide in order to identify reliable factors associated with the early diagnosis of pesticide-related diseases.

## Figures and Tables

**Table 1:** Characteristics of the selected datasets.

Dataset ID	Exposed (*N*)	Unexposed *(N)*	Platform	Sample Type	Pesticide	Ref
**Gene Expression**						
GSE30335	20	20	GPL570 [HG-U133_Plus_2] Affymetrix Human Genome U133 Plus 2.0 Array	Whole blood from exposed workers	Multiple exposure	N/A
GSE109513	81	9	GPL13158 [HT_HG-U133_Plus_PM] Affymetrix HT HG-U133+ PM Array Plate	HepG2—Liver cancer cells	Imazalil, Fenbuconazole, 2,4-D	[30]
GSE109511	81	9	GPL13158 [HT_HG-U133_Plus_PM] Affymetrix HT HG-U133+ PM Array Plate	HepaRG—Liver normal cells	Imazalil, Fenbuconazole, 2,4-D	[30]
GSE109509	81	9	GPL13158 [HT_HG-U133_Plus_PM] Affymetrix HT HG-U133+ PM Array Plate	MCF7—Breast cancer cells	Imazalil, Fenbuconazole, 2,4-D	[30]
GSE94521	30	20	GPL570 [HG-U133_Plus_2] Affymetrix Human Genome U133 Plus 2.0 Array	(hESC)-derived neural crest cells (NCC)—Normal Cells	Arsenic trioxide, Triadimefon, Cyproconazole, Geldanamycin, PBDE99, Trichostatin A	[31]
GSE35642	12	6	GPL96 [HG-U133A] Affymetrix Human Genome U133A Array	SK-N-MC—Brain cancer cells	Rotenone	[32]
**DNA Methylation**						
GSE90117	25	23	GPL13534 Illumina HumanMethylation450 BeadChip (HumanMethylation450_15017482)	Whole blood from child with or without prenatal pesticide exposure	Multiple exposures	[33]
GSE38646	21	6	GPL8490 Illumina HumanMethylation27 BeadChip (HumanMethylation27_270596_v.1.2)	K562—Hematological cancer cells	Diazinon, Dichlorvos, Chlorpyrifos, Terbufos, Fonofos, Phorate, Prathion	N/A
**miRNA Expression**						
GSE78805	11	4	GPL21545 Agilent-040157 Zebrafish_Human miRNA v18 [miRNA_ID version]	Zebrafish larvae	Atrazine	[34]

**Table 2:** Differentially expressed miRNAs after pesticide exposure.

miRNA ID	log2FC	*p*-Value
**Up-Regulated**		
hsa-miR-3149	2.613	0.0281
hsa-miR-4505	2.200	0.0333
hsa-miR-4687-3p	0.417	0.0039
hsa-miR-4758-5p	0.259	0.0133
hsa-miR-1268b	0.236	0.0484
**Down-Regulated**		
hsa-miR-455-3p	−0.116	0.0349
hsa-miR-23a-3p	−0.124	0.0499
hsa-miR-30e-3p	−0.131	0.0363
hsa-miR-210	−0.155	0.0471
hsa-miR-4261	−0.181	0.0499
hsa-miR-324-3p	−0.222	0.0051
hsa-miR-29b-3p	−0.233	0.0246
hsa-miR-181c-5p	−0.363	0.0385
hsa-miR-3907	−0.412	0.0003
hsa-miR-101-3p	−0.414	0.0058
hsa-miR-3610	−2.262	0.0204
hsa-miR-10b-3p	−2.323	0.0220
hsa-miR-4515	−2.462	0.0480
hsa-miR-4313	−2.545	0.0146
hsa-miR-126-3p	−2.945	0.0002

**Table 3:** Molecular pathways altered by the five up-regulated miRNAs after pesticide exposure.

N.	KEGG Pathway	*p*-Value	#Genes	#miRNAs	Genes
1	Fatty acid degradation (hsa00071)	8.79E-04	3	3	ADH4, HADHA, EHHADH
2	TGF-beta signaling pathway (hsa04350)	8.79E-04	13	3	ID2, ROCK1, SMAD2, THBS1, PPP2CA, SMURF2, SMAD3, BMP8B, SKP1, SMAD4, GDF6, SP1, BMPR1A
3	Glycosphingolipid biosynthesis-ganglio series (hsa00604)	1.67E-02	4	2	HEXB, ST3GAL1, ST3GAL2, ST6GALNAC6
4	Gap junction (hsa04540)	1.67E-02	12	4	PRKCA, SOS2, TJP1, LPAR1, SOS1, GJD2, GUCY1A2, GJA1, MAP3K2, GRB2, PDGFRB, ADCY6
5	Cell adhesion molecules (CAMs) (hsa04514)	3.23E-02	21	3	WWC2, NRCAM, CDH2, NEGR1, SPN, SDC3, CD6, CNTNAP2, NRXN1, CLDN22, HLA-F, CD34, CADM1, CD226, NRXN3, VCAN, NLGN2, NLGN1, SDC2, PVRL3, ITGAL
6	Fatty acid elongation (hsa00062)	3.71E-02	2	2	HADHA, ELOVL7
7	Thyroid hormone synthesis (hsa04918)	3.71E-02	8	2	PRKCA, ATP1B2, CREB3L3, TSHR, CREB3L1, TG, SLC5A5, ADCY6
8	Amphetamine addiction (hsa05031)	3.71E-02	12	4	PRKCA, CREB3L3, CAMK4, CALM3, SIRT1, SLC18A2, GRIN3B, PDYN, CREB3L1, GRIN2A, GRIA3, GRIN2B

**Table 4:** Molecular pathways altered by the down-regulated miRNAs after pesticide exposure.

N.	KEGG Pathway	*p*-Value	#Genes	#miRNAs
1	ECM–receptor interaction (hsa04512)	3.63E-19	38	8
2	Proteoglycans in cancer (hsa05205)	4.37E-13	92	9
3	Viral carcinogenesis (hsa05203)	3.72E-12	94	9
4	Adherens junction (hsa04520)	3.74E-12	46	9
5	Renal cell carcinoma (hsa05211)	4.81E-07	40	8
6	Chronic myeloid leukemia (hsa05220)	6.31E-07	43	8
7	Pancreatic cancer (hsa05212)	1.40E-06	38	7
8	Hippo signaling pathway (hsa04390)	1.40E-06	58	9
9	Focal adhesion (hsa04510)	1.40E-06	100	9
10	p53 signaling pathway (hsa04115)	4.46E-06	41	9
11	Fatty acid biosynthesis (hsa00061)	7.57E-06	3	7
12	Colorectal cancer (hsa05210)	7.57E-06	36	8
13	FoxO signaling pathway (hsa04068)	2.57E-05	67	9
14	PI3K-Akt signaling pathway (hsa04151)	2.89E-05	141	9
15	Glioma (hsa05214)	3.83E-05	33	8
16	Small cell lung cancer (hsa05222)	5.04E-05	46	8
17	Lysine degradation (hsa00310)	5.78E-05	22	7
18	Protein processing in endoplasmic reticulum (hsa04141)	7.18E-05	76	9
19	Nonsmall cell lung cancer (hsa05223)	1.08E-04	29	7
20	Pathways in cancer (hsa05200)	1.85E-04	157	9
21	Fatty acid metabolism (hsa01212)	1.87E-04	16	8
22	Sphingolipid signaling pathway (hsa04071)	1.87E-04	53	9
23	Cell cycle (hsa04110)	2.12E-04	61	9
24	Prostate cancer (hsa05215)	2.53E-04	46	8
25	Estrogen signaling pathway (hsa04915)	3.85E-04	45	9
26	Regulation of actin cytoskeleton (hsa04810)	4.42E-04	88	9
27	Endocytosis (hsa04144)	4.93E-04	86	9
28	Endometrial cancer (hsa05213)	7.39E-04	27	8
29	Neurotrophin signaling pathway (hsa04722)	1.07E-03	56	9
30	Ubiquitin mediated proteolysis (hsa04120)	1.35E-03	63	8
31	mTOR signaling pathway (hsa04150)	2.96E-03	32	9
32	RNA transport (hsa03013)	4.16E-03	68	9
33	Thyroid hormone signaling pathway (hsa04919)	4.16E-03	54	9
34	Signaling pathways regulating pluripotency of stem cells (hsa04550)	4.58E-03	56	9
35	Melanoma (hsa05218)	5.40E-03	33	7
36	Progesterone-mediated oocyte maturation (hsa04914)	5.40E-03	41	8
37	Prolactin signaling pathway (hsa04917)	5.78E-03	33	7
38	Phosphatidylinositol signaling system (hsa04070)	5.78E-03	35	9
39	Acute myeloid leukemia (hsa05221)	7.93E-03	27	8
40	Central carbon metabolism in cancer (hsa05230)	9.70E-03	30	9
41	Transcriptional misregulation in cancer (hsa05202)	1.29E-02	67	8
42	Oocyte meiosis (hsa04114)	1.44E-02	49	9
43	AMPK signaling pathway (hsa04152)	1.54E-02	52	9
44	TGF-beta signaling pathway (hsa04350)	1.54E-02	33	9
45	Inositol phosphate metabolism (hsa00562)	1.90E-02	26	8
46	Insulin signaling pathway (hsa04910)	2.15E-02	58	9
47	Biotin metabolism (hsa00780)	2.32E-02	1	1
48	Fc gamma R-mediated phagocytosis (hsa04666)	2.52E-02	39	8
49	Dorso-ventral axis formation (hsa04320)	2.66E-02	15	7
50	Axon guidance (hsa04360)	3.83E-02	45	9
51	Steroid biosynthesis (hsa00100)	3.93E-02	8	6
52	mRNA surveillance pathway (hsa03015)	4.71E-02	40	9

## Data Availability

The data analyzed in the present study are publicly available on the GEO DataSets database (https://www.ncbi.nlm.nih.gov/gds, accessed on 15 August 2021). The results obtained here are available from the corresponding author and available upon reasonable request.

## Supplementary Materials

The following are available online at https://www.mdpi.com/article/10.3390/ijerph18168697/s1, Figure S1: Dysregulated genes with concordant expression levels in the three datasets of normal cells and the two datasets of tumor cells. The fold change values are expressed as log2 of fold change.
