# Supplementary material for: Genetic and Epigenetic Alterations Induced by Pesticide Exposure: Integrated Analysis of Gene Expression, microRNA Expression, and DNA Methylation Datasets
Source: Int J Environ Res Public Health. 2021 Aug 17;18(16):8697. doi: 10.3390/ijerph18168697 (PMC8394939; doi:10.3390/ijerph18168697)
Supplement: Supplementary file 1 [file ijerph-18-08697-s001.zip › ijerph-1319488-supplementary.pdf]

A

Gene ID	Tumor Cell Line		
	MCF7	SK-N-MC	HepG2
	GSE109509	GSE35642	GSE109513
OBSL1	-0.1936	-0.0995	-0.234
HPS1	-0.1567	-0.0984	-0.231
SLC44A1	0.2344		0.193
MMP28	0.1731		0.256
MAP4K1	-0.1939	-0.187	-0.352
KLF9		-0.42	-0.220
BRD4		-0.635	-0.215
FANCC	-0.2289	-0.595	-0.178
GTSE1	-0.2574	-0.292	
ITPR2	-0.2215	-0.336	
TFDP1	-0.2095	-0.109	
PCDH7	-0.3876	-0.326	
MLX	-0.1487	-0.115	
PEX2	-0.1480	-0.159	-0.189

B

Gene ID	Normal Cell Line	
	hESC	HepaRG
	GSE94521	GSE109511
HPS1	0.289	0.310
SLC44A1	0.226	0.146
ATP6V0E1	0.323	0.347
MED27	0.267	0.149
HIPK2	0.312	0.161
PAWR	0.278	0.290
PTP4A3	-0.180	-0.280
WSB1	0.181	0.311
ADD2	0.244	0.311
ZMAT3	0.318	0.176
UBE2D2	0.114	0.233
RUBCN	0.243	0.219
ITPR2	0.258	0.582
PTBP1	-0.149	-0.118
PKN2	0.168	0.240
FANCA	-0.184	-0.206
MLX	-0.135	-0.290
ADCY1	-0.218	-0.257

Figure S1. Dysregulated genes with concordant expression levels in the three datasets of normal cells and the two datasets of tumor cells. The fold change values are expressed as log2 of fold change. Up-regulated genes are highlighted with red scale boxes while down-regulated genes are high-lighted with blue scale boxes.
